# Supplementary figures and images for: Role of Stomatal Conductance in Modifying the Dose Response of Stress-Volatile Emissions in Methyl Jasmonate Treated Leaves of Cucumber (Cucumis Sativa)
Source: Int J Mol Sci. 2020 Feb 4;21(3):1018. doi: 10.3390/ijms21031018 (PMC7038070; doi:10.3390/ijms21031018)

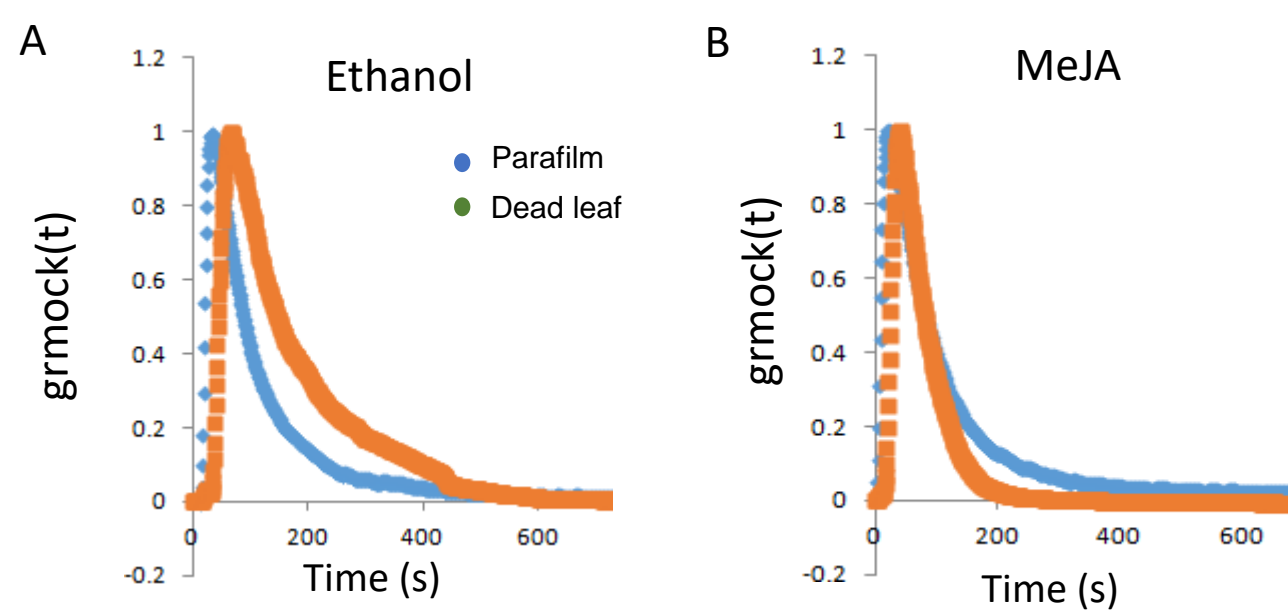

Figure S1

Supplement: Supplementary file 1 [file ijms-21-01018-s001.pdf]
